# Comparison of thyroid disease prevalence in patients with celiac disease and controls 

**Published:** 2020

**Authors:** Parastoo Baharvand, Maryam Hormozi, Asghar Aaliehpour

**Affiliations:** 1 *Department of Social Medicine, School of Medicine, Lorestan University of Medical Sciences, Khorramabad, Iran*; 2 *Department of Biochemistry, School of Medicine, Lorestan University of Medical Sciences, Khorramabad, Iran *; 3 *Department of Pathology, School of Medicine, Lorestan University of Medical Sciences, Khorramabad, Iran*

**Keywords:** Celiac disease, Thyroid disorders, Autoimmune, Epidemiology

## Abstract

**Aim:**

This study aimed to investigate the prevalence of thyroid disease (TD) in untreated CD patients and to evaluate the effect of gender and age on its prevalence.

**Background:**

Celiac disease (CD) is a form of intestinal malabsorption syndrome which is closely related to endocrine disorders, especially autoimmune thyroid disease and diabetes**.** The prevalence of TD is possibly high among patients with CD which necessitates the need for screening for TD among them.

**Methods:**

This comparative cross-sectional study was conducted on 288 consecutive untreated patients with CD (mean age, 27.9±14) and 250 controls (mean age, 29.01±13.15) referred for endoscopy in a hospital located in Iran. Thyroid function was evaluated by measuring T3, T4, and TSH levels using ELISA technique, and testing anti-thyroperoxidase (anti-TPO) antibodies through electrochemiluminescence method. Data analysis was conducted in SPSS v.22 software using descriptive statistics and chi-squared test.

**Results:**

Thyroid disease prevalence was 4-fold higher in patients than in controls (13.6% vs. 3.2%, p<0.05). Hypothyroidism was diagnosed in 30 patients and 7 controls, while hyperthyroidism was observed in 9 patients and in one control. Chi-squared test results reported a significant difference in TD prevalence between patients and controls based on gender and age (p<0.05). In both groups, women were signiﬁcantly more affected than men, and the TD prevalence was higher in younger patients compared to adults.

**Conclusion:**

There was a strong association between thyroid dysfunction and CD. In this regard, it is necessary to screen patients for TD.

## Introduction

 Celiac disease (CD) is an immune-mediated enteropathy. It is triggered by exposure to gluten (1-4), though some previous studies in literature are conflicting (3-5). The CD is known by “gastrointestinal symptoms, macroscopic and microscopic changes in the small bowel mucosa, malabsorption, and a wide range of extraintestinal manifestations” (6). It is largely related to human leukocyte antigen (HLA) genotypes (DQ2 and DQ8) (7, 8). The prevalence of CD varies within different countries. Previous studies have reported its prevalence as about 1% in the general population in European countries as well as in Iran (9, 10). There is ample evidence of a strong association between CD and several immune mediated diseases such as autoimmune thyroid disease, Sjogren’s syndrome, type 1 diabetes mellitus, Addison, Turner and Down syndromes, primary biliary cirrhosis, inflammatory bowel diseases, and autoimmune adrenal failure (2, 3, 11-18). Among these, autoimmune thyroid disease is a common organ specific autoimmune disorder whose prevalence is 10–12% in the general population worldwide (2, 19). 

The pathogenesis of co-existent autoimmune thyroid disease and CD is unknown (12), though epidemiological studies suggest a common genetic background for these T cell–mediated diseases (3, 20, 21). Several studies have reported a higher prevalence of thyroid disease (TD) in adults with CD than in the general population (22-23). In a study in Italy, TD was 3-fold higher in untreated CD adults, while the prevalence of TD in children with CD was 26.2% (24). In Sweden, its prevalence was reported 10.8% (25), and children with CD had a higher prevalence of TD (26). Meloni et al. (27) showed that TD was strongly associated with CD in Sardinian children and its prevalence was 10.5%. In Saleem et al.’s study conducted in Ireland, the prevalence of TD in adults with CD was 7% (28). Toumi et al. (29) in evaluating the frequency of anti-thyroid antibodies in Tunisian patients with CD reported that its frequency in CD patients was 8.3%. In the study by Mainardi et al. (30), the prevalence of CD in patients with TD was 2%. The prevalence of TD in patients with CD has increased suggesting that CD patients should be screened for TD (27). Untreated TD can result in marked morbidity in CD (31). Considering the previously reported associations between TD and CD, and the importance of TD treatment in CD patients, and given that, to our best knowledge, almost no studies have been carried out in Iran on screening the prevalence of TD in these patients, this study attempted to investigate its prevalence in patients with and without CD in Iran. Moreover, since age and gender are factors that affect thyroid function, we also evaluated their effect on TD prevalence. 

## Methods

This is a comparative cross-sectional study. The statistical population consisted of all patients diagnosed with CD (based on biopsy reports; i.e. showing villous atrophy in duodenum/ jejunum biopsies or with Marsh histopathology grade type 3) plus clinical symptoms such as recurrent abdominal discomfort, bloating, and diarrhea, and no warning signs who were referred consecutively to endoscopy department of Shohada-ye Ashayer Hospital in Khorramabad, Iran during 2016-2017 (n=288). For initial screening of CD in patients, their anti-tissue transglutaminase (anti-tTG) IgA antibody was measured via Enzyme-Linked Immunosorbent Assay (ELISA) method by considering the upper normal limit of 20 U/mL. The serum tTG-IgA level was determined by nephelometry and binding assay kit with a normal IgA range of 70-400 mg/dL. In case of low IgA antibody titer, anti-tTG IgG antibody was measured by ELISA method with a normal range of < 20 u/mL. Since they were newly diagnosed with CD, they were still untreated. All of them were enrolled in te study (census sampling) and assigned to the CD group. There were also 250 samples with abdominal pain and dyspepsia according to endoscopy and biopsy test results and had no CD. They were all assigned to the control group (consecutive sampling). None of them had a past or present history of thyroid disease and thyroid drug use (entry criteria). The exclusion criteria for the patients were the presence of disease that may affect thyroid function (any factor that affects the pituitary or hypothalamus itself such as tumors, trauma, infiltrative diseases, and metabolic diseases), the use of medication that may affect serum blood levels (e.g. antiepileptic and anticonvulsant drugs such as phenytoin, phenobarbital, valproate, carbamazepine, protease inhibitors which are antiviral drugs as well as nonsteroidal anti-inflammatory drugs, rifampin, and furosemide), any systemic disease, inflammatory bowel disease, pregnant and lactating women, abdominal mass, and unwillingness to participate in the study. For the controls, exclusion criteria were the recent use of antibiotics, antiepileptic and anticonvulsant or anti-depressants, protease inhibitors, rifampin, furosemide, as well as unwillingness to participate in the study.

In order to screen the TD in patients, levels of tri‐iodothyronine (T3), thyroxine (T4), and thyroid‐stimulating hormone (TSH) were measured using ELISA method. The standard criterion for the presence of thyroid dysfunction was TSH level. In this regard, patients with TD were subdivided into three groups: Euthyroid, hypothyroid and hyperthyroid. Euthyroidism was defined as a TSH level within 0.5–4.5 mIU/L (normal), while for hypothyroidism, TSH>4.5 uµ/ml, and for hyperthyroidism as TSH<0.5 mIU/L was considered. Moreover, the level of anti-thyroperoxidase (anti-TPO) antibodies in the blood samples was measured using electrochemiluminescence (ECL) method. Autoimmunity was diagnosed when anti-TPO antibody level was >34 IU/mL. The collected data were analyzed in SPSS v.22 by presenting descriptive statistics (mean, standard deviation, frequency, percentage) and using chi-squared test for comparing the TD prevalence between groups in terms of age and gender. The significance level was set at 0.05 (P<0.05). 

## Results

In the CD group, there were 102 males and 186 females with a mean age of 27.9±14 years (87 were <20 years and 201 were >20 years). The control group consisted of 186 females and 102 males with a mean age of 29.01±13.15 years (75 <20 years of age and 175 >20 years old). As reported in Table 1, overall, TD was found in 39 of 288 patients (13.6%) and 8 of 250 controls (3.2%). In the patients, 30 (10.4%) had hypothyroidism, 9 (3.2%) hyperthyroidism, 249 (86.4%) thyroid function, and 5 (1.7%) positive anti-TPO test result. In the control group, 7 (2.8%) had hypothyroidism, one (0.4%) hyperthyroidism, 242 (96.8%) normal thyroid function, and one positive anti-TPO result (0.4%). The difference between patients and controls in terms of TD prevalence was statistically significant (p<0.05).

Considering gender, among patients diagnosed with TD, 21 females (11.3%) and 9 males (8.8%) had hypothyroidism, while hyperthyroidism was found in 8 females (4.3%) and one male (1.1%). Among controls, 6 females (3.6%) and one male (1.2%) had hypothyroidism, while hyperthyroidism was diagnosed in one female (0.7%) and in no males. As can be seen, in both groups, the TD prevalence was higher in females than in males. As provided in Table 2, the results showed a significant difference in TD prevalence between females (p=0.021<0.05) and males (p=0.035<0.05) with and without CD.

Regarding age, of 30 patients with hypothyroidism, 12 (13.7%) had age less than 20 years and 18 (8.9%) were older than 20 years; of 9 patients with hyperthyroidism, 2 (2.4%) aged less than 20 years and 7 (3.6%) were older than 20 years. Further, of 7 controls with hypothyroidism, one (1.4%) had an age less than 20 years and 6 (3.5%) more than 20 years, while the one (1.4%) with hyperthyroidism aged less than 20 years. As shown in Table 3, it was observed that there was a significant difference between two age groups (<20 and >20 years) of samples with and without CD (p<0.05).

**Table 1:** Chi-squared test results for comparing demographic factors and TD frequency in patients and controls

Variables		Celiac disease; N(%)	Control; N(%)	P-value
Gender	Female	186(64.6)	165(66)	0.834
	Male	102(35.4)	85(34)	
Age	<20 years	87(30.2)	75(30)	0.725
	>20 years	201(69.8)	175(70)	
TD	Hypothyroid	30(10.4)	7(2.8)	0.001
	Hyperthyroid	9(3.2)	1(0.4)	0.001
	Euthyroid	249(86.4)	242(96.8)	0.317
Positive anti-TPO		5(1.7)	1(0.4)	0.03
Total		288(100)	250(100)	

**Table 2:** Chi-squared test results for comparing TD frequency in both groups based on gender

Sex		Celiac disease; N(%)	Control; N(%)	P-value
Female	Hypothyroid	21(11.3)	6(3.6)	0.021
	Hyperthyroid	8(4.3)	1(0.7)	
	Euthyroid	157(84.4)	158(95.7)	
Total		186(100)	165(100)	
Male	Hypothyroid	9(8.8)	1(1.2)	0.035
	Hyperthyroid	1(1.1)	-	
	Euthyroid	92(90.1)	84(98.8)	
Total		102(100)	85(100)	

**Table 3:** Chi-squared test results for comparing TD frequency in both groups based on age

Age			Celiac disease; N(%)	Control; N(%)	P-value
<20 years		Hypothyroid	12(13.7)	1(1.4)	.001
		Hyperthyroid	2(2.4)	1(1.4)	
		Euthyroid	73(83.9)	73(97.2)	
Total			87(100)	75(100)	
>20 years	Hypothyroid		18(8.9)	6(3.5)	.001
	Hyperthyroid		7(3.6)	-	
	Euthyroid		176(87.5)	169(96.5)	
Total			201(100)	175(100)	

## Discussion

There are several studies reporting an association between CD and other autoimmune diseases especially thyroid disease (3, 12, 13, 18, 22), though there are some conflicts (3-5, 30). This study attempted to screen the prevalence of TD in the study population (adults and children) with CD. The results highlighted that patients with CD are at risk for developing TD with an overall 4-fold higher frequency than in controls. Thyroid disease was diagnosed in 39 out of 288 patients (13.6%) and in 8 of 250 controls (3.2%). There was a significant difference between patients and controls in terms of gender and age. In both groups, women were signiﬁcantly more affected than men (15% vs. 10% in patients, and 4.3% vs. 1.2% in controls). This might be attributed to the hormonal and genetic differences as well as their effect on autoimmune diseases. This is in agreement with the results of Sategna-Guidetti et al. (22), Meloni et al. (27), and Ludvigsson et al. (32). Furthermore, the prevalence of TD was higher in younger patients compared to adults (16% vs.12.5%). This result is consistent with the findings of Elfström et al. (13), Roy et al. (15) and Elfström et al. (23). In the present study, the prevalence of TD in younger patients (aged <20 years) was 16%. In the study by Guariso et al. (18), 27% children had autoimmune disease vs. 1% among the controls. Ansaldi et al. (24) reported the prevalence of TD as 26.2% among Italian CD children (mean age 8.5 years) vs. 10% in control subjects. Also, Van der Pals et al. (26) reported it as 7% among Swedish patients (12 years old children). In another study, Meloni et al. (27) reported a TD prevalence of 10.5% in children with CD (mean age 10.5 years). Among older patients (aged>20 years), the prevalence rate in our study was reported 12.5%. Sategna-Guidetti et al. (22) reported the overall TD among adult patients as 30.3%. In the study by Saleem (28), TD was found in 7% of adult patients, while in Toumi et al.’s study (29) on Tunisian adult patients, 4.8% had TD. The discrepancy may be because of differences in TSH levels and the diagnosis kits was used.

Based on the results of the current study, 10.4% of patients had hypothyroidism and 3.2% hyperthyroidism, while among controls, hypothyroidism was found in 2.8% and hyperthyroidism in 0.4%. In the study by Sategna-Guidetti et al. (22), hypothyroidism was diagnosed in 12.9% of Italian patients and 4.2% controls, and there was no difference regarding hyperthyroidism. Elfström et al. (23) examined the risk of thyroid disease in Swedish patients with CD, and reported that CD was associated with hypothyroidism (hazard ratio = 4.4) and hyperthyroidism (hazard ratio=2.9). Da Silva Kotze et al. (33) reported increased prevalence of TD (hypothyroidism, 19.2%; and hypothyroidism, 21.2%) in Brazilian patients with CD. In Sun et al.’s study, the prevalence of hypothyroidism among patients with CD was significantly increased compared with that in healthy groups, but the prevalence of hyperthyroidism was not significantly elevated (25). The association between CD and TD may justify the increased autoimmune diseases seen in CD (4), and can be explained by the presence of shared genetic factors. Human HLAs (DQ2 and DQ8) are both common in thyroid disease and CD, and patients with overlapping disease are often HLA DQ2 positive (23). The different results between our study and above-mentioned studies can be related to the differences in sample size, study place, type of study, and the diagnostic methods and techniques used.

In our study, anti-TPO antibodies were found in the blood samples of 5 (1.7%) patients and one control. Various studies have indicated that there is a time interval between the diagnosis of CD and thyroid dysfunction in patients with CD. The time period has been different in different studies. In the present study, anti-TPO antibodies were tested in both groups immediately after blood sampling (at the same time with TD prevalence screening). In the study by Kalyoncu and Urganci (34), anti-TPO test became positive in 16.4% of the pediatric patients 2 to 3 years after the diagnosis of CD. They observed clinical hypothyroidism only in 27.2% of CD patients with positive antithyroid antibodies. Wessels et al. (35) suggested that patients with CD do not require screening for thyroid disorders up to 5 years after the diagnosis of CD. Canova et al. (36) observed that there was a 5-year interval between the diagnosis of CD and thyroid disorders, while according to Elfström et al. (23), the time interval was 9 years. According to studies, it seems that the average time of screening for thyroid disorders is at least 5 years following the diagnosis of CD in patients.

Thyroid dysfunction has a close association with CD. Thus, screening for TD in patients with CD is necessary. There is probably a higher propensity for thyroid autoimmunity. A longitudinal follow-up is recommended with a larger sample size to screen the exact frequency of TD and other disorders associated with CD. Moreover, the impact of gluten-free diets on the treatment of these disorders and their possible complications in patients should be evaluated.

## References

[1] Green PH, Cellier C (2007). Celiac disease. N Engl J Med.

[2] Sharma BR, Joshi AS, Varthakavi PK, Chadha MD, Bhagwat NM, Pawal PS (2016). Celiac autoimmunity in autoimmune thyroid disease is highly prevalent with a questionable impact. Indian J Endocrinol Metab.

[3] Kurien M, Mollazadegan K, Sanders DS, Ludvigsson JF (2016). Celiac disease increases risk of thyroid disease in patients with type 1 diabetes: a nationwide cohort study. Diabetes Care.

[4] Ventura A, Magazzù G, Greco L (1999). Duration of exposure to gluten and risk for autoimmune disorders in patients with celiac disease. Gastroenterology.

[5] Sategna-Guidetti C, Solerio E, Scaglione N, Aimo G, Mengozzi G (2001). Duration of gluten exposure in adult coeliac disease does not correlate with the risk for autoimmune disorders. Gut.

[6] Horwitz A, Skaaby T, Kårhus LL, Schwarz P, Jørgensen T, Rumessen JJ (2015). Screening for celiac disease in Danish adults. Scand J Gastroenterol.

[7] Fasano A (2005). Clinical presentation of celiac disease in the pediatric population. Gastroenterology.

[8] Barker JM, Liu E (2008). Celiac disease: pathophysiology, clinical manifestations, and associated autoimmune conditions. Adv Pediatr.

[9] Moradniani M, Sabzevari ZM, Aaliehpour A, Baharvand P (2017). Demographic features and high prevalence of celiac disease in patients with Irritable Bowel Syndrome in Khoram Abad, Lorestan. Govaresh.

[10] Mustalahti K, Catassi C, Reunanen A, Fabiani E, Heier M, McMillan S (2010). The prevalence of celiac disease in Europe: results of a centralized, international mass screening project. Ann Med.

[11] Collin P, Kaukinen K, Välimäki M, Salmi J (2002). Endocrinological disorders and celiac disease. Endocr Rev.

[12] Ch’ng CL, Jones MK, Kingham JG (2007). Celiac disease and autoimmune thyroid disease. Clin Med Res.

[13] Elfström P, Sundström J, Ludvigsson JF (2014). Systematic review with meta‐analysis: associations between coeliac disease and type 1 diabetes. Aliment Pharmacol Ther.

[14] Ventura A, Ronsoni MF, Shiozawa MBC, Dantas-Corrêa EB, Canalli MHBdS, Schiavon LdL (2014). Prevalence and clinical features of celiac disease in patients with autoimmune thyroiditis: cross-sectional study. Sao Paulo Med J.

[15] Roy A, Laszkowska M, Sundström J, Lebwohl B, Green PH, Kämpe O (2016). Prevalence of celiac disease in patients with autoimmune thyroid disease: a meta-analysis. Thyroid.

[16] Teixeira LM, Nisihara R, Utiyama SRdR, Bem RSd, Marcatto C, Bertolazo M (2014). Screening of celiac disease in patients with autoimmune thyroid disease from Southern Brazil. Arq Bras Endocrinol Metabol.

[17] Denham JM, Hill ID (2013). Celiac disease and autoimmunity: review and controversies. Curr Allergy Asthma Rep.

[18] Guariso G, Conte S, Presotto F, Basso D, Brotto F, Visona Dalla Pozza L (2007). Clinical, subclinical and potential autoimmune diseases in an Italian population of children with coeliac disease. Aliment Aliment Pharmacol Ther.

[19] Vanderpump MP (2011). The epidemiology of thyroid disease. Br Med Bull.

[20] Jabri B, Sollid LM (2009). Tissue-mediated control of immunopathology in coeliac disease. Nat Rev Immunol.

[21] Duntas LH, Orgiazzi J, Brabant G (2011). The interface between thyroid and diabetes mellitus. Clin Endocrinol.

[22] Sategna-Guidetti C, Volta U, Ciacci C, Usai P, Carlino A, De Franceschi L, Camera A, Pelli A, Brossa C (2001). Prevalence of thyroid disorders in untreated adult celiac disease patients and effect of gluten withdrawal: an Italian multicenter study. Am J Gastroenterol.

[23] Elfström P, Montgomery SM, Kämpe O, Ekbom A, Ludvigsson JF (2008). Risk of thyroid disease in individuals with celiac disease. J Clin Endocrinol Metab.

[24] Ansaldi N, Palmas T, Corrias A, Barbato M, D'altiglia MR, Campanozzi A (2003). Autoimmune thyroid disease and celiac disease in children. J Pediatr Gastroenterol Nutr.

[25] Sun X, Lu L, Yang R, Li YB, Shan L, Wang Y (2016). Increased Incidence of Thyroid Disease in Patients with Celiac Disease: A Systematic Review and Meta-Analysis. Plos One.

[26] Van der Pals M, Ivarsson A, Norstrom F, Hogberg L, Svensson J, Carlsson A (2014). Prevalence of thyroid autoimmunity in children with celiac disease compared to healthy 12-year olds. Autoimmune Dis.

[27] Meloni A, Mandas C, Jores RD, Congia M (2009). Prevalence of Autoimmune Thyroiditis in Children with Celiac Disease and Effect of Gluten Withdrawal. J Pediatr.

[28] Saleem A (2012). Adult coeliac disease in Ireland: a case series. Ir J Med Sci.

[29] Toumi D, Mankai A, Belhadj R, Ghedira-Besbes L, Jeddi M, Ghedira I (2008). Thyroid-related autoantibodies in Tunisian patients with coeliac disease. Clin Chem Lab Med.

[30] Mainardi E, Montanelli A, Dotti M, Nano R, Moscato G (2002). Thyroid-related autoantibodies and celiac disease: a role for a gluten-free diet?. J Clin Gastroenterol.

[31] Hakanen M, Luotola K, Salmi J, Laippala P, Kaukinen K, Collin P (2001). Clinical and subclinical autoimmune thyroid disease in adult celiac disease. Dig Dis Sci.

[32] Ludvigsson JF, Lebwohl B, Kampe O, Murray JA, Green PH, Ekbom A (2013). Risk of Thyroid Cancer in a Nationwide Cohort of Patients with Biopsy-Verified Celiac Disease. Thyroid.

[33] Da Silva Kotze LM, Nisihara RM, da Rosa Utiyama SR, Piovezan GCC, Kotze LR (2006). Thyroid disorders in Brazilian patients with celiac disease. J Clin Gastroenterol.

[34] Kalyoncu D, Urganci N (2015). Antithyroid antibodies and thyroid function in pediatric patients with celiac disease. Int J Endocrinol.

[35] Wessels MM, van Veen II, Vriezinga SL, Putter H, Rings EH, Mearin ML (2016). Complementary Serologic Investigations in Children with Celiac Disease Is Unnecessary during Follow-Up. J Pediatr.

[36] Canova C, Pitter G, Ludvigsson JF, Romor P, Zanier L, Zanotti R, Simonato L (2016). Celiac Disease and Risk of Autoimmune Disorders: A Population-Based Matched Birth Cohort Study. J Pediatr.

